# Use of HRP-2-based rapid diagnostic test for *Plasmodium falciparum *malaria: assessing accuracy and cost-effectiveness in the villages of Dielmo and Ndiop, Senegal

**DOI:** 10.1186/1475-2875-9-153

**Published:** 2010-06-04

**Authors:** Alioune Badara Ly, Adama Tall, Robert Perry, Laurence Baril, Abdoulaye Badiane, Joseph Faye, Christophe Rogier, Aissatou Touré, Cheikh Sokhna, Jean-François Trape, Rémy Michel

**Affiliations:** 1 Unité d'Epidémiologie des Maladies Infectieuses, Institut Pasteur de Dakar, BP 220 - Dakar, Sénégal; 2 President's Malaria Initiative, Dakar, Sénégal; 3 UR077, Institut de Recherche pour le Développement, Dakar, Sénégal; 4 Unit d'Immunologie, Institut Pasteur de Dakar, Sénégal; 5 Unité de Recherche en Biologie et Epidémiologie Parasitaires, Institut de Recherche Biomédicale des Armées - Antenne de Marseille, URMITE - UMR6236, Marseille, France

## Abstract

**Background:**

In 2006, the Senegalese National Malaria Control Programme (NMCP) has recommended artemisinin-based combination therapy (ACT) as the first-line treatment for uncomplicated malaria and, in 2007, mandated testing for all suspected cases of malaria with a *Plasmodium falciparum *HRP-2-based rapid diagnostic test for malaria (RDT(Paracheck^®^). Given the higher cost of ACT compared to earlier anti-malarials, the objectives of the present study were i) to study the accuracy of Paracheck^® ^compared to the thick blood smear (TBS) in two areas with different levels of malaria endemicity and ii) analyse the cost-effectiveness of the strategy of the parasitological confirmation of clinically suspected malaria cases management recommended by the NMCP.

**Methods:**

A cross-sectional study was undertaken in the villages of Dielmo and Ndiop (Senegal) nested in a cohort study of about 800 inhabitants. For all the individuals consulting between October 2008 and January 2009 with a clinical diagnosis of malaria, a questionnaire was filled and finger-prick blood samples were taken both for microscopic examination and RDT. The estimated costs and cost-effectiveness analysis were made considering five scenarios, the recommendations of the NMCP being the reference scenario. In addition, a sensitivity analysis was performed assuming that all the RDT-positive patients and 50% of RDT-negative patients were treated with ACT.

**Results:**

A total of 189 consultations for clinically suspected malaria occurred during the study period. The sensitivity, specificity, positive and negative predictive values were respectively 100%, 98.3%, 80.0% and 100%. The estimated cost of the reference scenario was close to 700€ per 1000 episodes of illness, approximately twice as expensive as most of the other scenarios. Nevertheless, it appeared to us cost-effective while ensuring the diagnosis and the treatment of 100% of malaria attacks and an adequate management of 98.4% of episodes of illness. The present study also demonstrated that full compliance of health care providers with RDT results was required in order to avoid severe incremental costs.

**Conclusions:**

A rational use of ACT requires laboratory testing of all patients presenting with presumed malaria. Use of RDTs inevitably has incremental costs, but the strategy associating RDT use for all clinically suspected malaria and prescribing ACT only to patients tested positive is cost-effective in areas where microscopy is unavailable.

## Background

Malaria is a major cause of morbidity and mortality and the large, round numbers that delineate its burden have now become familiar. In 2006, WHO estimated that 3.3 billion persons were at risk of malaria infection of whom 1.2 billion were at high risk, mostly in Africa (49%). Of the estimated one million annual deaths due to malaria, approximately 91% of them were thought to occur in Africa and 85% in children under five years of age [1]. In Senegal, in 2006, malaria was the first cause of morbidity and mortality and was estimated to be responsible for approximately 35% of the consultations in health care facilities and 8,000 annual deaths (total estimated population 12 million inhabitants). However, in many endemic countries patients are only clinically diagnosed and a small proportion of malaria cases are tested, owing to a lack of diagnostic capabilities. Therefore, there is considerable uncertainty surrounding the estimates of the number of cases and deaths, mainly in the African region, and any attempt to establish the number of malaria cases globally is subject to argument [2]. At the clinic level, treatments are often given presumptively to patients presenting with fever or other symptoms compatible with malaria. The emergence and spread of resistance to chloroquine and other anti-malarial drugs have had a dramatic impact on the evolution of malaria mortality [3], especially in Africa [4] and have prompted to change to more expensive therapeutic combinations. In 2006, the Senegalese National Malaria Control Programme (NMCP) has recommended the use of artemisinin-based combination therapy (ACT) as the first-line treatment for uncomplicated malaria. These combinations are highly effective but overall much more expensive than previous regimens [5]. In this context of increasing direct costs, rational therapeutic approach against malaria has become essential and there is a need to limit anti-malarial treatment to laboratory-confirmed malaria only.

Therefore, as recommended by the World Health Organization (WHO), rapid diagnostic tests for malaria (RDT) offer this opportunity. They have the advantage of being simple to perform, easy to interpret, requiring minimal infrastructure and thus are particularly indicated where microscopy, still considered as the gold standard, is not available. In order to overcome the problems of availability of diagnostics the NMCP started in 2007 to provide health care facilities with a rapid diagnostic test for malaria (RDT). Different types of RDTs based on immunochromatographic antigen capture are marketed. According to WHO, they must be capable of reliably detecting 100 parasites/μl (0.002% parasitaemia) from all *Plasmodium spp*. with a sensitivity of 100% and of giving rapid results (15 to 20 minutes) [6]. The most commonly used RDT target is a glycoprotein called HRP-2 (Histidine Rich Protein 2), an antigen specific for *Plasmodium falciparum *secreted from erythrocytes infected with rings, trophozoites, schizonts and immature gametocytes. HRP-2-based tests have been shown accurate in detecting *P. falciparum *infections [7]. However, their specificity is a cause for concern, particularly in areas of intense malaria transmission due to persistence of HRP2 antigens from previous infections [8,9]. *Plasmodium *lactate dehydrogenase (pLDH) is the other major targeted antigen. This enzyme is expressed at high levels in all blood-stages of the parasite, and all four human malarial parasites produce a unique pLDH activity. In addition, pLDH antibodies can also be used to detect *Plasmodium knowlesi *[10], a monkey malaria parasite capable of infecting humans. The level of pLDH in the blood is directly linked to the level of parasitaemia. Furthermore, LDH do not persist in blood after clearance of parasitaemia and may be a good marker for both initial diagnosis of *Plasmodium *infections and while following anti-malarial efficacy [11]. Lastly, some RDTs detect the fructose-1,6-diphosphate aldolase, an enzyme of the *Plasmodium *glycolytic pathway [12]. Monoclonal antibodies produced against *Plasmodium *aldolase are pan-specific and have been used in combination with HRP-2, making tests capable of distinguishing an infection with *P. falciparum *from that due to non- *P. falciparum*.

An earlier study in Senegal showed that HRP-2-based tests may be especially useful in areas where *P falciparum *is predominant and where skilled microscopy is not readily available [13]. In 2007, with help from the Global Fund, the NMCP implemented a new diagnostic and treatment guidelines based on the testing of all suspect cases, with Paracheck^® ^(Orchid Biomedical Systems, Goa, India), an HRP-2-based test with the objective to achieve the revised targets set by African Heads of State in Abuja in 2000, of 80% population coverage by 2010 [14]. The main objectives of requiring parasitological confirmation of suspected cases are to avoid costs of treatment of non-malarial fevers by ACT, to better understand the rates of disease and death due to malaria in Senegal, to reduce unnecessary exposure to potential adverse effects of ACT and to prevent the spread of parasite resistance to these drugs, already described in Southeast Asia [15-17]. Since this introduction the country has experienced a dramatic decrease in malaria-associated morbidity and mortality (proportional morbidity from 33.6% to 5.6% and proportional mortality from 18.2% to 7.1% between 2006 and 2008 (NMCP, unpublished data), with some of the decrease likely due to vector control measures put into place.

The objectives of this study were to evaluate, under field conditions, the accuracy of Paracheck^® ^compared to TBS in two areas with different levels of malaria endemicity and to conduct a cost-effectiveness analysis of the diagnostic and treatment strategy recommended by the Senegalese NMCP, taking into account the public health impact of the introduction of ACT and RDTs.

## Methods

### Study site and population

**Figure 1:**
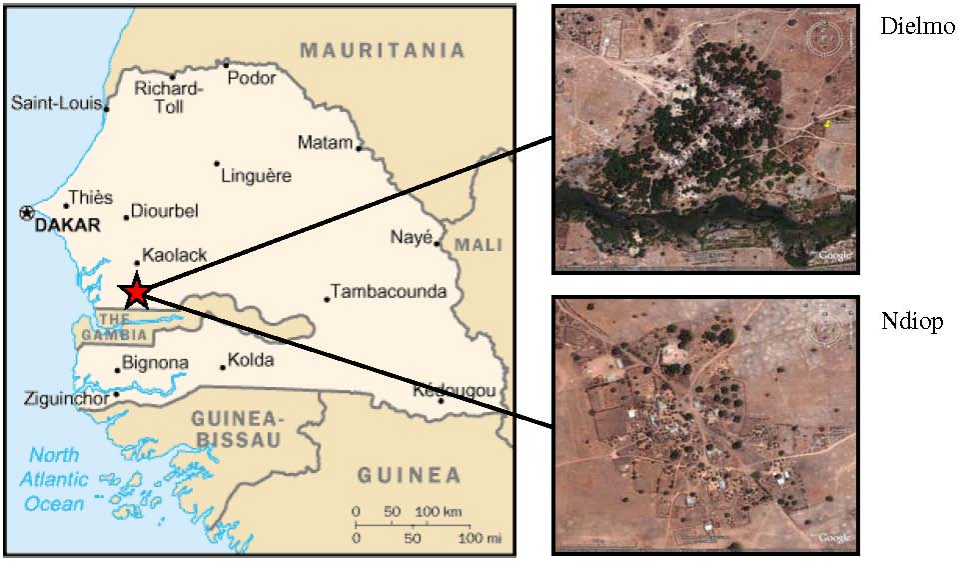
**Senegal map showing the geographical localization of Dielmo and Ndiop villages (Sources Wikitravel.org and Google earth)**.

The study was undertaken in the villages of Dielmo and Ndiop, located in a sahelo-soudanian region of Senegal approximately 280 km south-east of Dakar, and 10 km north of the Gambian border (Figure 1). The Dielmo-Ndiop project, initiated in 1990 Dielmo and 1993 in Ndiop respectively is a longitudinal study of host-parasite relationships and the mechanisms of protective immunity against malaria. The ongoing longitudinal epidemiological and entomologic follow-up at this study site has been described in detail elsewhere [18]. In April 2008, the population of Dielmo was 460, of whom 424 (92.2%) were enrolled in the longitudinal follow-up. In this village, malaria is highly endemic, with intense perennial transmission and an entomologic inoculation rate (EIR) varying around 200 infected bites/person/year [19]. This high level of transmission is the result of the permanent presence of a small river next to the village whose banks host anopheline larval development sites year round. The village of Ndiop, only 5 km far from Dielmo, has 410 inhabitants of whom 374 (91%) were enrolled in the study. In this village, malaria transmission is mesoendemic, with around 30 infected bites/person/year, and highly seasonal, occurring during the rainy season, from July to October [20]. For years, the annual number of laboratory confirmed malaria cases from these two villages has varied from 500 to 700.

The longitudinal follow-up consists of a daily visit to actively search for suspect cases, the presence of a field research dispensary, a laboratory, and permanent health staff in each village. Each suspect case is confirmed using TBS and, if confirmed, treated with ACT and followed systematically with TBS at day 4 and 7.

### Study design

A cross-sectional study was undertaken at the end of the rainy season, when a peak of malaria incidence is usually observed. Included in the study were all individuals from the Dielmo-Ndiop longitudinal follow-up, consulting at one of the two field research dispensaries during the study time period, consenting to participate in this specific study on RDTs (parent or legal guardian for the children), and presenting with a clinically-suspected malaria (fever or suspicion of fever, cephalgia, diarrhoea or vomiting) requiring a TBS. Excluded were individuals presenting with severe malaria or any other severe illness and those declining to give consent for this study.

### Data collection and statistical analysis

For all individuals consulting at the dispensary with a clinical diagnosis of malaria, finger-prick blood samples were taken both for microscopic examination, in accordance with the cohort protocol. For each patient included, a RDT was done and a standardized questionnaire was filled by the health personnel. All RDTs were carried out and interpreted before reading the blood smear, following manufacturers' instructions, by technicians present in each village. A second control reading of the slides was done by an experienced microscopist from the Institut de Recherche pour le Développement (IRD) in Dakar who was masked to the RTD and field slide results. All TBS readings followed a standard protocol, [21] with a total of 200 microscopic oil-immersion fields examined (approximately 0.5 μL of blood); which is more than it is often the case in comparative studies, and the parasite density estimated from the ratio of the total number of trophozoites in the 200 fields to the average number of leucocytes per microscopic field. Gametocytes were recorded separately and not included in the asexual parasite count.

The term "episode of illness" refers to any clinically-suspected cases of malaria according to the health worker's perception while "malaria" refers only to episodes of illness confirmed by positive control readings of the blood smears. The sensitivity, specificity, positive and negative predictive values of RDTs were calculated using the TBS as the reference test, as recommended by WHO [22]. Confidence intervals of the accuracy indicators were calculated using the exact method. Costing assessment was undertaken from the NMCP perspective by considering the non-subsidized costs of RTDs and anti-malarials for outpatients in public health facilities. The age distribution of case was taken into account given the treatment dosage varying according to age groups. Costs were calculated using only direct costs related to the use of RDTs and ACT. Neither indirect costs (loss of productivity, infrastructures, other treatments ...) nor those common to the studied strategies were assessed.

One objective of using RDTs is to limit as much as possible the prescription of ACT to people who actually have *P. falciparum *parasitaemia. For this reason, the primary measure of effectiveness was the proportion of patients that would have been correctly managed (proportion of malaria attacks treated with ACT + proportion of non-malaria illness with no anti-malarial treatment), the reference being the result of the blood smear. The secondary measure of effectiveness was the proportion of all malaria attacks that would have been treated with ACT. Full compliance of health care providers with tests results was assumed, what is consistent with some published studies [23-26].

Five scenarios were considered:

1. **Presumptive treatment of all the febrile episodes of illness (body temperature ≥ 38°C)**: scenario frequently found in the literature and which supposes that all febrile episodes of illness are treated with ACT [27,28].

2. **Presumptive treatment of some episodes of illness according to the healthcare provider's feeling**: here, was taken into account the attitude that the healthcare provider would have adopted in the absence of any malaria diagnostic tool. It is a more pragmatic approach, nearer to the field reality than the first scenario. It is also the attitude which was prevailing before the RDTs were deployed in Senegal.

3. **Treatment of all episodes of illness RDT positive**: it corresponds to the current recommendations of the Senegalese NMCP, *i.e. *performing a RDT in clinically suspected malaria case and prescribing ACT if tested positive. This was the scenario of reference.

4. **Treatment of febrile episodes of illness with positive RDT**: in this scenario, a RDT is performed in case of body temperature > 38°C only and ACT prescribed in the event of positive RDT. This scenario, which can constitute an alternative to the current recommendations of the NMCP, is also found in the literature [27,29].

5. **Treatment of all children under six and treatment of all episodes of illness RDT positive for patients over six**: a scenario recommended by the WHO 2006 treatment guidelines.

However, other studies have demonstrated that at least 50% of malaria-negative patients are treated with anti-malarial drugs [30,31]. For this reason, costs of scenarios 3 to 5 were also estimated using a more realistic figure for adherence to TDR result, assuming that 50% of malaria-negative patients were treated with anti-malarials (sensitivity analysis).

Epi-info Version 3.5.1 (CDC, Atlanta, GA, USA) was used for data entry and Stata Version 10 (StataCorp, College Station, Texas, USA) for statistical analysis. Cost-effectiveness analysis results were displayed using the diagram proposed by Drummond [32].

### Ethical approval

The Dielmo-Ndiop program, which was used as support for this work was approved by the Ministry of Health and the Prevention's National Ethics Committee in 2006 (Comité National d'Ethique pour la Recherche en Santé). A separate approval was also obtained from this committee for the present study. Written informed consents were obtained from all the participants in the study (or guardians of children under 15 years of age).

## Results

### Descriptive analysis

A total of 189 consultations for clinically suspected malaria were recorded between October 2008 and January 2009, 122 in Dielmo and 67 in Ndiop. The proportion of females was statistically higher than males (59.8%, P < 0.01). The median age of the sample was 5.7 years (Q1-Q3 interval: 1.2 - 18.2 years). The distribution by age group was 51.8% under six years, 16.4% from six to 13 years and 31.8% older than 13 years. Age groups were the same as those used for estimating treatment doses. The distribution of the sample population by sex and age was not statistically different between the two villages.

### Clinical symptomatology

The most frequent symptom was a history of fever or a feeling that the body is hot (152 cases - 80.4%). A body temperature > 38°C was recorded in 94 cases (49.7%) at the time of the consultation. Headache and weakness were notified in 57.7% and 49.0% of cases, respectively.

### Presumptive anti-malarial treatment

In half the cases (95/189) the health care provider stated that he would have given ACT on the basis of the clinical picture and in the absence of means of definitive diagnosis of malaria (blood smear or RDT). In the univariate analysis, body temperature > 38°C, diarrhoea, anorexia and an age < 6 years were associated with a presumptive treatment by ACT. The proportion of episodes of illness, which would have benefited from ACT decreased statistically with increased age (Chi² for linear trend: P < 0.001).

### Parasitological diagnosis of malaria

A total of 189 TBS and 189 RDT were performed at the initial consultation. Twelve (6.4%) malaria attacks were confirmed by positive blood smears (8/122 in Dielmo and 4/67 in Ndiop). No discrepancy (positive or negative) was found in the parasite density between the first reading at the time of the initial consultation and the control reading. Rapid testing was positive for 15 cases. False positive RDT results were found in three boys of six months, five years and 11 years of age living in Dielmo, the village with higher malaria endemicity. The five year-old had been treated for slide-positive malaria 14 days prior to inclusion in the study and the 11 year-old showed the presence of *P. falciparum *gametocytes on the TBS. The six-month old child had no clear explanation for the positive RDT results.

### Clinical symptomatology of confirmed cases

A body temperature > 38°C was recorded at the time of the initial consultation for six (50%) of the 12 cases of slide-positive malaria (range 38.2 - 39.8°C). In three (25%) cases of slide-positive malaria, no fever or history of fever was recorded. In these three cases, the main symptom was intense cephalgia. Cases from the two villages were not significantly different by gender, age, temperature, or parasite density.

### Accuracy of Paracheck^® ^compared to expert microscopy

As the test characteristics for RDTs done in Dielmo and Ndiop were not significantly different, the data from the two villages were pooled. Table 1 shows the results of the test characteristics of Parachek^®^, in comparison with TBS readings, by village and for the whole sample. Follow-up RDT and TBS were performed at D4 and D7 for 10 of the 12 confirmed malaria cases. Of these 10, while the follow-up TBS readings from D4 were all negative, six (60%) had a persistent HRP-2 antigenaemia at D4 and four (40%) at D7. Five (83%) of the six positive RDT at D4 and all four positive RDT at D7 were from Dielmo.

**Table 1:** Accuracy assessment of Paracheck^®^, by village and for the whole sample

RDT characteristic	Village		Total sample (N = 189)	
	
	Dielmo (n = 122)	Ndiop (n = 67)	(%)	95%CI*
Sensitivity	100	100	100	[73.5 - 100]
Specificity	97.4	100	98.3	[95.1 - 99.6]
Positive Predictive Value	72.7	100	80.0	[51.9 - 95.7]
Negative Predictive Value	100	100	100	[97.9 - 100]

* 95%CI : 95% Confidence Interval (exact method)

### Cost-effectiveness analysis

#### Costs

The non-subsidized cost of a treatment course with artesunate-amodiaquine varies according to age: 0.47€ for children from 1 to 6 years, 0.87€ for children from seven to 13 years and 1.73€ for both children of more than 13 years and adults. Paracheck^® ^tests cost 0.61€ each. Table 2 gives the estimated costs for 1,000 cases managed according to each scenario, assuming that the age distribution is the same as that observed in the study sample. Except for scenario 5, the estimated cost of the reference scenario was close to 700€ per 1,000 episodes of illness, roughly double the three other scenarios, where the estimated cost was around 350 € per 1,000 episodes of illness. The approximate increase in costs for the reference scenario ranged between 330 and 350€ per 1,000 episodes of illness.

**Table 2:** Costs comparison (in Euros) between the four scenarios, estimated on the 189 episodes of illness (October 2008 and January 2009).

	Age distribution (n)	Cost of RDT & ACT	Total cost (study sample)	Cost per 1000 EI*	cost difference with scenario3 (per 1000 EI) & 95%CI
Scenario 1: Presumptive treatment of all the febrile EI*					
(number of RDT = 0, number of ACT course = 94)					
RDT cost		**0**			
ACT cost		**66,68**	**66,68**	**352,8**	**- 336,3 [-320,0 ; -355,0]**
1-6 y	70	32,9			
7-13 y	9	7,83			
> 13 y	15	25,95			

Scenario 2: Presumptive treatment of EI* according to the healthcare provider's feeling					
(number of RDT = 0, number of ACT course = = 95)					
RDT cost		**0**			
ACT cost		**68,35**	**68,35**	**361,6**	**- 327,5[ -311,2 ; -346,2]**
1-6 y	68	31,96			
7-13 y	12	10,44			
> 13 y	15	25,95			

Scenario 3: Treatment of all EI* RDT positive					
(number of RDT = 189, number of ACT course = 15 [12 ; 19])					
RDT cost		**115,29**		**689,10**	
ACT cost		**14,95**	**130,24**	**[672,8 ; 707,8]**	**Reference**.
1-6 y	6	2,82			
7-13 y	4	3,48			
> 13 y	5	8,65			

Scenario 4: Treatment of febrile EI* RDT positive					
(number of RDT = 94, number of ACT course = 8 [6 ;11])					
RDT cost		**57,95**		**335,3**	
ACT cost		**5,42**	**63,37**	**[328,0 ; -346,0]**	**- 353,8 [-326.8 ; -379,8]**
1-6 y	6	2,82			
7-13 y > 13 y	1 1	0,87 1,73			

**Scenario 5: Treatment of all children **≤ **6 and of all episodes of illness RDT positive for patients > 6**					
(number of RDT = 91, number of ACT course = 107 [105 ; 109])					
RDT cost		**55.51**		**601,6**	
ACT cost		**58.19**	**113.7**	**[587,8 ; 615.3]**	**-87.5 [-57.5 ; -120.0]**.
1-6 y	98	46.06			
7-13 y	4	3,48			
> 13 y	5	8,65			

* EI = Episode of illness

#### Effectiveness

Scenarios 1 and 2, i.e. those based on the presumptive treatment of suspected malaria, would have resulted in the correct management of only half of the episodes of illness while scenarios 3 and 4, based on the use of the RDT, would have resulted in correct management of nearly all of the episodes (98.4% and 95.8%, respectively). The effectiveness of scenario 5 was estimated between these two values (58.2%). However, considering the secondary measure of effectiveness, scenario 4 would have resulted in ACT treatment for only half of the malaria attacks. Table 3 shows the proportion of episodes of illness with adequate management for each scenario.

**Table 3:** Proportion of correctly managed episodes of illness or malaria attacks for the four scenarios considered.

	Malaria attack (N = 12)		Non-malaria attack (N = 177)		Total EI* (N = 189)		
	
	Treated with ACT	Non-treated with ACT	Non-treated with ACT	Treated with ACT	Episodes of illness with adequate management		
	
	n *(%)*	n *(%)*	n *(%)*	n *(%)*	n	(%)	[IC95%]
**Scenario 1**	**6 (50.0)**	6 (50.0)	**89 (50.3)**	88 (49.7)	95	(**50.3)**	[42.9 ;57.6]
**Scenario 2**	**8 (66.7)**	4 (33.3)	**90 (50.8)**	87 (49.2)	98	(**51.8)**	[44.5 ;59.2]
**Scenario 3**	**12 (100)**	0 (00.0)	**174 (98.3)**	3 (1.7)	186	(**98.4)**	[95.4 ;99.6]
**Scenario 4**	**6 (50.0)**	6 (50.0)	**175 (98.9)**	2 (1.1)	181	(**95.8)**	[91.8 ;98.1]
Scenario 5	**12 (100)**	0 (00,0)	**98 (55.4)**	79 (44.6)	110	(58.2)	[50.8 ;65.3]

* EI = Episode of illness

#### Cost-effectiveness analysis

**Figure 2:**
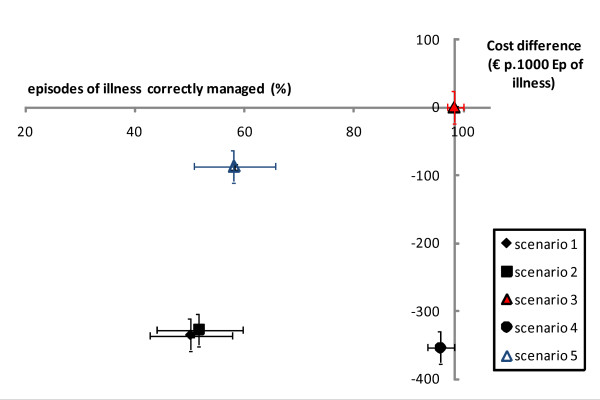
**Cost-effectiveness analysis comparing four scenarios (primary measure of effectiveness: proportion of episodes of illness with adequate management)**.

**Figure 3:**
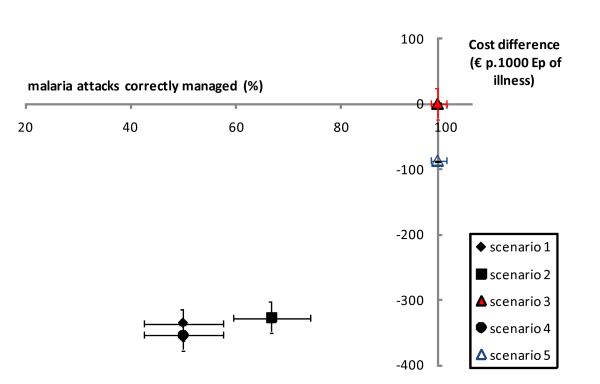
**Cost-effectiveness analysis comparing four scenarios (secondary measure of effectiveness: proportion of malaria attacks that would have benefited from ACT)**.

Figure 2 shows the cost-effectiveness analysis for the primary measure of effectiveness (proportion of episodes of illness correctly managed). All four scenarios lie in the left lower quadrant of the graph (less expensive but also less effective than the reference). Compared to the reference scenario, the others were neither dominant (less costly and more effective) nor dominated (more costly and less effective). Based on the primary measure of effectiveness, scenario 4 seemed to be a reasonable alternative, as it was half as expensive and equally effective as the reference scenario. However, considering the secondary measure of effectiveness, the proportion of malaria attacks that would have benefited from an anti-malarial treatment (Figure 3), scenario 4 would adequately manage only half the malaria attacks and thus would be no more effective than the presumptive treatment strategies. Still considering this secondary measure of effectiveness, scenario 5 appeared to be less costly while as effective as the reference scenario.

#### Sensitivity analysis

Costs of scenarios 3 to 5 were estimated assuming that all the RDT-positive patients and 50% of RDT-negative patients were treated with ACT. Costs of each scenario were estimated to be around 1.5 fold higher than when assuming full compliance to the test results (Table 4). Under this assumption, the proportions of episodes of illness correctly managed were estimated of 52.4% and 54.3% for scenarios 3 and 4, respectively. This proportion dropped to 29.6% in scenario 5.

**Table 4:** Sensitivity analysis (costs of scenarios 3 to 5 estimated on the 189 episodes of illness assuming that 50% of malaria-negative patients were treated with anti-malarials (October 2008 and January 2009).

	RDT Positive	RDT Negative	EI treated	Cost of RDT & ACT	Total cost (study sample)	Total cost per 1000 EI*
Scenario 3 modified: Treatment of all EI* RDT positive & 50% of EI RDT negative						
(number of RDT = 189, number of ACT course = 102)						
RDT cost				**115.29**		
ACT cost				**95.89**	**211.18**	**1117.35**
1-6 y	6	92	52	24.4		
7-13 y	4	27	17.5	15.2		
> 13 y	5	55	32.5	56.2		

Scenario 4 modified: Treatment of febrile EI* RDT positive & 50% of febrile EI RDT negative						
(number of RDT = 94, number of ACT course = 51)						
RDT cost				**57.95**		
ACT cost				**36.05**	**94.00**	**497.35**
1-6 y	6	64	38	17.86		
7-13 y	1	8	5	4.35		
> 13 y	1	14	8	13.84		

Scenario 5 modified: Treatment of all children ≤ 6 and of all EI* RDT positive & 50% of EI RDT negative for patients > 6						
(number of RDT = 91, number of ACT course = 88)						
RDT cost				**55.51**		
ACT cost				**117.51**	**173.02**	**915.45**
1-6 y			98	46.06		
7-13 y	4	27	17.5	15.22		
> 13 y	5	55	32.5	56.23		

## Discussion

### Accuracy of rapid testing

The accuracy of rapid testing was very good compared to TBS. At D0, sensitivity, specificity, positive and negative predictive values were of 100%, 98.3%, 80% and 100%, respectively. These results are consistent with other published studies showing that the sensitivity and specificity of HRP-2-based tests usually are > 90% for *P. falciparum *[9,33,34] and that they can be a useful tool in the management of patients with suspected malaria, especially where microscopic diagnosis is not available [35,36]. However, the results observed can vary according to transmission intensity [37]. In zones of high transmission, the specificity of these tests can be lower. Studies performed In the Democratic Republic of Congo in children aged 6-59 months [38] and in a hyperendemic region of Uganda [39] found 52% to72% specificity for the HRP-2 based test they evaluated. A lower specificity was also found in Dielmo where all three false positive results were recorded, though only one child had a history of a prior malaria episode. The specificity of these tests is usually higher in low malaria transmission areas like Ndiop, similar to what was found in an urban area in Tanzania [40]. However, in low endemicity areas, some authors described a lower sensitivity, related to a low parasitaemia rate [41-43], which was not observed in the study in Ndiop. The persistence of HRP-2 antigenaemia after effective treatment is thought to be the most frequent cause of false positive results for HRP-2-based RDT [44]. In the present study as well, 60% of RDT carried out on D4 and 40% carried out on D7 were still positive, corresponding to a specificity of the RDT of 40% at D4 and 60% at D7. Other studies showed that 35% to 61% of patients still had HRP-2 antigenaemia 14 days after treatment [45,46] and that this antigenaemia could persist up to 35 days after treatment [38,39].

Another cause of false positive results with HRP-2-based tests found in Dielmo is the presence of early gametocytes [47,48]. The third case of false positive RDT results was a six-month old child who had neither recent history of fever nor any other obvious reason for a positive result. It is possible that this "false positive" result was due to a temporary and asymptomatic parasitaemia during the weeks preceding the test in a child still protected by maternal antibodies or to a parasitaemia lower than the threshold of microscope detection [37,44].

### Cost-effectiveness analysis

Through scenario 2, this study took into account the attitude of the healthcare personnel whether or not to prescribe ACT in clinically suspected cases of malaria when estimating costs. Although studies assume that only febrile patients are considered to have malaria, in reality almost 20% of anti-malarials are given to patients who do not have a history of fever [49]. While this study only took account of direct costs related to the use of the ACT and RDT, other studies have taken into account other parameters like the costs of microscopy (materials and staff time), the cost of any second line treatment and antibiotics, or health outcomes in terms of disability-adjusted life years averted [25,34]. Unlike Zikuzooka's findings [28], the present study did not show that using RDTs could be cost-saving and it is likely that the cost of the additional benefit brought by RDTs may be higher than many countries can afford without external assistance or lower RDT prices [24].

This study confirmed the high operational accuracy of Paracheck^® ^but also shows the increased risk of false-positive results as transmission increases, probably related to persistent antigenemia after treatment, that could lead to misdiagnoses and thus of inappropriate prescriptions of ACT. However, in Senegal zones of high endemicity remain limited and the most representative epidemiologic setting is the strictly seasonal transmission of Ndiop. Under the scenarios based on rapid testing (3 and 4), ACT would have been prescribed in only 1.7% to 11.3% of the non-malaria episodes of illness, while under the scenarios based on clinical diagnosis (1 and 2) this proportion would reach nearly 50%, consistent with Luxemburger's findings [50]. This analysis also confirms that RDTs are good tools for malaria diagnosis where microscopy is unavailable and for reducing the risk of clinical failure with presumptive treatment [42]. In addition, RDTs make it possible to collect more reliable data on malaria epidemiology and its proportional morbidity and mortality. Malaria represented only 3.2% of the episodes of illness and 6.4% of the cases of fever in the present study. In other studies, this RDT positivity rate ranges from 6 to 52% of clinically diagnosed malaria cases in areas of low-moderate transmission [8] and can reach 87% in high-transmission areas [23]. It is, therefore, necessary to encourage and train healthcare personnel to consider other diseases besides malaria as the cause of fever.

The cost of the scenario corresponding to the current recommendations of the NMCP in Senegal was estimated around 700€ per 1,000 episodes of illness, approximately twice as expensive as the others scenarios considered, except for scenario 5. Nevertheless, it still appeared to us cost-effective as it ensured the correct diagnosis and treatment of 100% of malaria attacks and an adequate management of 98.4% of episodes of illness. The other scenarios, while less costly, were also less effective. Scenario 4 was close to the reference scenario when considering the primary measure of effectiveness, but it would have resulted in the correct diagnosis and treatment of only 50% of malaria cases and thus cannot be recommended for ethical and public health reasons. Scenario 5 could be a possible alternative to the reference scenario when the primary measure of effectiveness is considered.

However, the sensitivity analysis of the present study demonstrated that full compliance of health care providers with RDT results was required in order to avoid severe incremental costs.

Studies undertaken in sub-Saharan Africa have shown that, when ACT is used, confirming cases with RDTs was cost-effective compared to presumptive treatment when the parasite prevalence was below 62% and may help to improve management of non-malaria febrile illness, particularly bacterial infections [25,51]. However, the specificity of RDTs and results of cost-effectiveness analyses should be interpreted considering the level of endemicity, season, age of patients or presence/absence of fever during consultation [8], and whether patients with RDT negative results are prescribed anti-malarial as has been frequently shown to be the case [30,31]. Given the greater burden of malaria in Africa, economic analyses can be very sensitive to small changes in the cost of treatment or diagnosis [24]. In the future RDTs should become more cost-effective as the Senegalese NMCP completes its plan to replace the current ACT with a better tolerated but more expensive one and as the risk of malaria is reduced [28].

### Limitations

A limitation of this study was the low malaria incidence during recruitment. This incidence was the lowest observed since the Dielmo-Ndiop cohort follow-up was begun in 1990. Such a drop in incidence, observed following the deployment of ACT and vector control measures in Senegal and described elsewhere [52], would only improve the cost-effectiveness of RDT use. Another limitation was that only costs of anti-malarial treatment and diagnostic testing were included and not indirect costs such as those related to management of patients initially wrongly diagnosed, productivity losses or treatment costs of non-malaria cases.

## Conclusions

According to the results of the present study, strategies based on presumptive treatments of clinically suspected malaria, or febrile illnesses, or on performing RDT in case of fever only lead to a high level of misdiagnosis.

In settings where microscopy is unavailable, using RDT can lead to a significant reduction in the overprescription of anti-malarials [53] Though the use of RDTs has inevitable incremental costs, such an investment offers a more promising strategy to deal with increasing costs of therapy driven by drug resistance [34]. This strategy results in a rational use of ACT and ensure their sustainability in limiting improper treatments leading to drug wastage and resistance [54]. In addition, confirming all cases with RDTs will provide more reliable data on malaria epidemiology.

The present study confirmed the benefits in promoting the use of RDTs in remote areas where microscopy is unavailable. However, it will be necessary to repeat these studies as malaria epidemiology changes and to test RDTs targeting both falciparum and non-falciparum parasites.

## Competing interests

The authors declare that they have no competing interests.

## Authors' contributions

RM, ABL ATall initiated the study and participated in the design of the study. LB was the head of the Unit of Infectious Diseases Epidemiology when the study was conducted. AB participated in the field study. JF performed data management. ABL and RM performed the statistical analysis and interpreted data, with the help of CR.

The paper was essentially written by RM, with the input of ABL and RP, All authors read, critically revised and approved the manuscript.
